# Incidence of invasive Group B Streptococcal infection and the risk of infant death and cerebral palsy: a Norwegian Cohort Study

**DOI:** 10.1038/s41390-020-1092-2

**Published:** 2020-07-29

**Authors:** Maren Mynarek, Solveig Bjellmo, Stian Lydersen, Jan E. Afset, Guro L. Andersen, Torstein Vik

**Affiliations:** 1 Department of Clinical and Molecular Medicine, Norwegian University of Science and Technology, 7491 Trondheim, Norway; 2 Department of Obstetrics and Gynecology, Helse More og Romsdal HF, Aalesund, Norway; 3 Regional Centre for Child and Youth Health and Child Welfare, Department of Mental Health, PB 8905, MTFS, 7491 Trondheim, Norway; 4 Department of Medical Microbiology, St. Olavs Hospital, Trondheim University Hospital, Trondheim, Norway; 5 Vestfold Hospital Trust, The Cerebral Palsy Registry of Norway, PB 2168, 3103 Tønsberg, Norway

## Abstract

**Background:**

Group B Streptococcus (GBS) is the leading cause of invasive neonatal infection worldwide. In high-income countries mortality rates are 4–10%, and among survivors of GBS meningitis 30–50% have neurodevelopmental impairments. We hypothesized that invasive GBS infection was associated with increased risk of infant mortality and cerebral palsy (CP).

**Methods:**

All children born alive in Norway during 1996–2012 were included. Data were collected from three national registers. Invasive GBS infection during infancy was categorized into early-onset disease (EOD), late-onset disease (LOD), and very late-onset disease (VLOD). Primary outcomes were infant mortality and CP.

**Results:**

Invasive GBS infection was diagnosed in 625 children (incidence: 0.62 per 1000 live births; 95% confidence interval (CI): 0.57–0.67). The incidence of EOD was 0.41 (0.37–0.45), of LOD 0.20 (0.17–0.23), and of VLOD 0.012 (0.007–0.021). The annual incidence of LOD increased slightly. Among infected infants, 44 (7%) died (odds ratio (OR): 24.5; 95% CI: 18.0–33.3 compared with the background population). Among survivors, 24 (4.1%) children were later diagnosed with CP, compared with 1887 (0.19%) in the background population (OR: 22.9; 95% CI: 15.1–34.5).

**Conclusion:**

Despite a relatively low incidence of invasive GBS infection in Norway, the risk of death and CP remains high. Improvements in prevention strategies are needed.

**Impact:**

- During the first decade of the twenty-first century, invasive GBS disease in infancy is still associated with high mortality.
- Despite the overall low incidence of invasive GBS disease, the incidence of LOD increased during the study period.
- The finding that invasive GBS infection in the neonatal period or during infancy is associated with an excess risk of CP, comparable to the risk following moderate preterm birth and moderate low Apgar scores, adds to the existing literature.
- The results of this study emphasize the importance of adhering to guidelines and the need for better prevention strategies.

## Introduction

*Streptococcus agalactiae* (Group B streptococcus, GBS) remains a leading cause of invasive infection in neonates.^1^ Despite improvements in maternal and perinatal care, GBS infection is still associated with substantial mortality and morbidity.^2,3^ Invasive infection presenting within the first 7 days of life is classified as early-onset disease (i.e., EOD), while infection presenting between 7 and 89 days of life is classified as late-onset disease (i.e., LOD).

EOD often results from vertical transmission from the mother, either before or during delivery. Approximately 20–30% of pregnant women are colonized in the vagina and/or rectum, and ~50–70% of their newborns will be colonized. One to two percent of these children will develop invasive GBS infection.^4^

Administration of antibiotic treatment intrapartum can reduce vertical transmission during delivery. In the United States, and some European countries, all pregnant women are screened for GBS, and intrapartum antibiotic prophylaxis (IAP) is given if the women is colonized.^5^ The potential downside of this practice is that >30% of all newborns in the United States are exposed to intrapartum antibiotics.^6^ This practice may raise concerns regarding development of antibiotic resistance, as well as long-term consequences following disturbances of the establishment of the newborn microbiota.^7^ Another approach has therefore been applied by other countries, including Norway, the UK, New Zealand, the Netherlands, Denmark, and Sweden, where antibiotic prophylaxis is given only when specific risk factors for neonatal GBS disease are present.^5^

While the incidence of EOD has declined since the late 1990s in many countries such as the US and Australia,^1,6^ the incidence of LOD has remained stable, or even increased.^8–12^ Furthermore, invasive GBS infection is still associated with miscarriages, stillbirths, perinatal and neonatal mortality, and preterm labor, as well as significant infant morbidity and adverse long-term outcomes.^2^ In particular, it has been estimated that 30–50% of the survivors from GBS meningitis suffer from neurodevelopmental impairments (NDIs).^3^ The long-term neurodevelopmental outcomes of other invasive GBS infections are less well described.^3^

Cerebral palsy (CP) is the most common cause of motor disability in young children, affecting 2.4 per 1000 liveborn children in Norway.^13^ Recent studies have reported that perinatal mortality has continued to decrease in the general population, and even the prevalence of CP has decreased during the first decades of the twenty-first century.^14^ These improvements, which have been assigned to better antenatal, intrapartum, and postnatal care,^13^ may also have improved the outcome of invasive GBS infection.

Therefore, the aim of this study was to assess the burden of disease associated with invasive GBS infection in infancy. We hypothesized that invasive GBS infection was associated with an increased risk of mortality and CP, comparable to the risk of CP following premature birth before 32 weeks gestation, or following moderate asphyxia (i.e., Apgar <7), that is, 20–30-fold increased. We also hypothesized that children who were diagnosed with CP following invasive GBS infection in infancy would be more severely disabled and more often have spastic bilateral CP compared to other children with CP.

## Methods

Eligible to participate in this population-based cohort study were all children born alive in Norway from 1996 to 2012. Children born before gestational week 23 and later than week 43 were excluded.

Information on GBS disease was retrieved from the Norwegian Surveillance System for Communicable Diseases (MSIS) and combined with information from the Medical Birth Registry of Norway (MBRN) regarding maternal health before and during pregnancy, the course of the delivery and newborn health, as well as with neurodevelopmental information retrieved from the Cerebral Palsy Registry of Norway (CPRN).

The MSIS is a national registry for selected contagious diseases at the Norwegian Institute of Public Health. Since 1986 it has been compulsory to report invasive infections with GBS to MSIS. The MSIS receives these notifications in two supplementary ways. Hospital laboratories report information on any positive culture, PCR, or antigen test of GBS from a normally sterile body site, while clinicians are required to report all cases of invasive GBS infections in infants by completing a registration form, including basic demographic, epidemiological, and clinical information.

The MBRN records information on all births after 12 weeks of gestation in Norway. Registration is compulsory and data on all children born since 1967 have been registered. The register comprises some demographic information as well as information on various aspects of the pregnancy and birth, including maternal health before and during pregnancy, delivery, early intervention, and treatment of the newborn.

The CPRN is an informed consent-based national quality register recording information on all children with CP born since 1996. It contains detailed information about age at time of diagnosis, postnatal causes, CP subtypes, and additional difficulties, such as epilepsy, speech difficulties, and eating difficulties. The data are provided by neuropediatric habilitation centers and is recorded at 5 years of age when the diagnosis has been confirmed. The correctness and completeness of this register is well documented.^13^

Data from the three registers were linked using the 11-digit personal identification number unique for every Norwegian citizen.

The main exposure variable was invasive GBS infection in infancy (i.e., in children up to 1 year of age) confirmed by culture, PCR, or antigen test, as reported to the MSIS. The clinical diagnoses of sepsis, meningitis, and pneumonia were mainly based on the information from the MSIS with some modifications; in addition to those who were recorded with meningitis in the MSIS, we included children in this diagnostic group, if GBS had been cultured in their cerebrospinal fluid (CSF), even if the diagnosis meningitis was not recorded in the MSIS.

Moreover, we defined sepsis as present in all children with a positive blood culture with GBS, in the absence of a clinical diagnosis of meningitis or pneumonia, as well as in children with a clinical diagnosis of sepsis with a positive urine antigen test, or GBS isolated from lung aspirate, “other” or “unknown” material. The clinical diagnosis of pneumonia in the MSIS was not further modified. “Other invasive infections” included children with a positive GBS culture, PCR, or antigen test, and a clinical diagnosis of urinary tract infection, arthritis, or a diagnosis not further specified in MSIS. Based on age at onset, infections were categorized as early onset disease (EOD) when diagnosed between 0 and 6 days postpartum, late onset disease (LOD) when diagnosed between 7 and 89 days, and very late-onset disease (VLOD) when diagnosed between 90 and 365 days postpartum.

The primary outcomes were mortality during infancy (i.e., ≤12 months of life) and CP. We studied overall mortality as recorded in the MBRN as well as deaths assigned to GBS infection as recorded in the MSIS. The latter information was used to calculate case fatality rate (i.e., the proportion of children who died due to the GBS infection among children with invasive infection). CP was diagnosed and classified according to the definition recommended by the Surveillance of Cerebral Palsy in Europe.^15^ The CP diagnosis was validated at 5 years of age.

As secondary outcomes, we studied if invasive GBS infection in infancy was associated with any specific CP subtype (i.e., spastic bilateral and unilateral, dyskinetic, and ataxic CP), or with more severe gross and fine motor impairments, as well as with comorbidities, such as epilepsy, speech, and feeding difficulties. Gross motor function was classified into five levels according to the Gross Motor Function Classification System (GMFCS).^16^ Fine motor function was also classified into five levels, using the Manual Ability Classification System (MACS).^17^ In both classification systems, level I indicates the least severe and level V the most severe impairments. Speech ability was classified into four levels using the Viking speech scale, whereby level I indicates normal speech and level IV no understandable speech.^18^ Epilepsy was defined as present in children who were treated with antiepileptic drugs at the time of the 5-year registration in the CPRN. The presence of gastrostomy tube feeding was used to indicate severe eating difficulties.

Information on maternal age, parity, pre-eclampsia, pregnancy-induced hypertension, maternal diabetes mellitus, prelabor rupture of membranes (PROM), discolored/malodourous amniotic fluid, delivery method, gestational age, sex, multiple gestation, and birth weight was retrieved from the MBRN.

Gestational age in Norway is assessed using ultrasound examination around pregnancy week 18 in >80% of cases, while in the remaining pregnancies, gestational age is estimated based on the last menstrual period.

Newborns with a birth weight below −2 SD, adjusted for gestational age and sex according to Norwegian growth standards, were defined as being small for gestational age (SGA).^19^

### Statistical analysis

Differences in proportions between groups were analyzed using Pearson chi-squared test. For small samples, we used the unconditional Z-pooled test as recommended by Lydersen et al.^20^ Incidence per 1000 with 95% confidence intervals (CI) was calculated using the Wilson score as recommended by Fagerland et al.^21^ Incidence was calculated using the number of infants with invasive GBS infection in the respective year, divided by live births during the same year. Binary regression with identity link function and birth year as covariate was used to estimate time trends in the incidence of any GBS, EOD, LOD, and VLOD. We used the identity link function instead of log odds link function (which is used in logistic regression) because we regard the absolute risk and risk differences to be easier to interpret and clinically more meaningful than odds ratios (ORs) in this context. To study other trends, we used the linear-by-linear association test. Logistic regression was used to calculate OR with 95% CI as estimates of the relative risk for CP in children with GBS infection in infancy, compared with the reference group. Two-sided *P* values <0.05 were considered to indicate statistical significance, and 95% CIs were reported where relevant.

We also performed sensitivity analyses where we excluded children with GBS isolated from “other” or “unknown” material, lung aspirate, or where GBS was identified only through a positive urine antigen test. In these analyses, the excluded cases were included in the control group. We used Stata15 for binary regression and CIs for proportions, and IBM SPSS 25 was used for all other analyses.

### Ethics

The study was approved by the Regional Committee for Medical and Health Research Research Ethics in Central Norway (reference number 2011/754). Parents of children with CP have provided written informed consent allowing their children to be registered in the CPRN and to link data to the MBRN, as well as to other Norwegian health registers. The committee approved that an additional informed consent for this specific study was not required.

### Patient involvement

The association for persons with CP in Norway and their families (“CP foreningen”) has a representative in the advisory group of the CPRN where they also participate in the discussion of research questions. They were however not involved in the formulation of the research question addressed in this study, the design, or in the completion of the present study.

## Results

A total of 1,016,732 children were born in the study period. After exclusion of children born before 23 weeks and after 43 weeks of gestation, as well as stillborn children, the study population comprised 1,007,246 children.

**Fig. 1:**
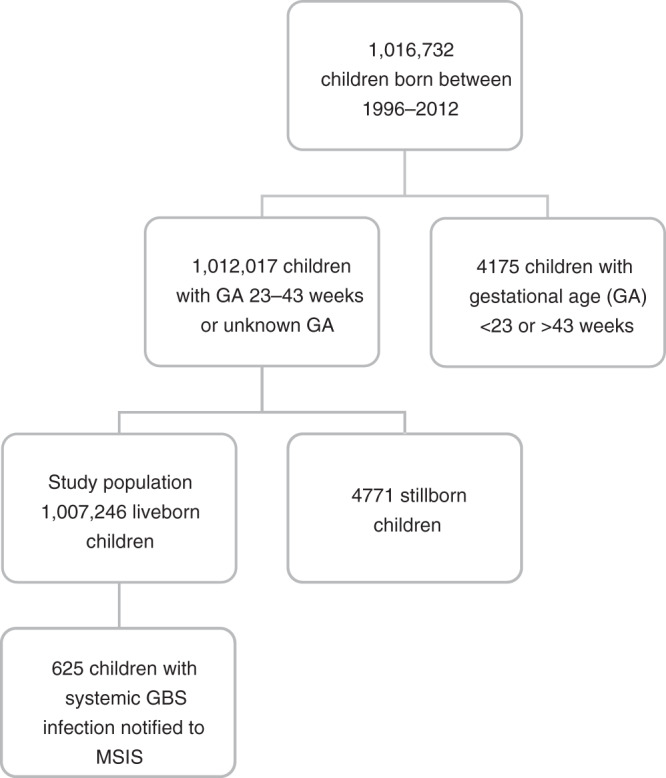
Flow chart of the study population. The flow chart shows the total number of children born in Norway between 1996 and 2012 (abstracted from the Medical Birth registry of Norway) and among these the study population after exclusion of children born before gestational age (GA) 23 weeks or later than GA week 43, and stillbirths. The number of children with invasive group B streptococcus (GBS) infection during the first year of life is also shown (abstracted from the Norwegian Surveillance System for Communicable Diseases).

**Fig. 2:**
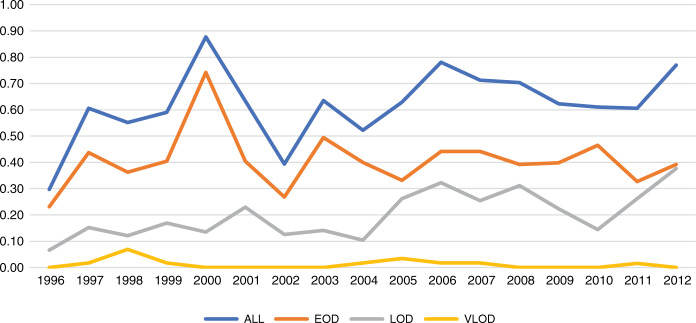
Trends in the incidence of invasive Group B Streptococcal (GBS) infection. The figure shows the the overall (blue line) trend in incidence (Y-axis) of invasive Group B Streptococcal (GBS) infection during the first year of life per 1000 live births in Norway during 1996–2012 (X-axis), as well as the trends for early-onset disease (EOD; orange line), late-onset disease (LOD; grey line), and very late-onset disease (VLOD; yellow line). The information was abstracted from the Norwegian Surveillance System for Communicable Diseases and the Medical Birth Registry of Norway. For calculations see Supplementary Table S1.

Of these, 625 children were diagnosed with an invasive GBS infection before 1 year of age (Fig. 1), resulting in a cumulative incidence of invasive GBS infections of 0.62 per 1000 live births (95% CI: 0.57–0.67) ranging between 0.30 in 1996 and 0.88 per 1000 in 2000 (Fig. 2 and Supplementary Table S1). The oldest child was 189 days at the time of diagnosis.

The incidence of EOD was 0.41 per 1000 (95% CI: 0.37–0.45), the incidence of LOD was 0.20 (95% CI: 0.17–0.23), while of VLOD the incidence was 0.012 (95% CI: 0.007–0.021). The incidence of invasive GBS infection increased by 0.011 per 1000 per year during the study period (95% CI: 0.001–0.021; *p* = 0.027). This increase was caused by an increase in LOD of 0.012 per 1000 live births per year (95% CI: 0.006–0.017, *p* < 0.0001) while the incidences of EOD and VLOD remained stable.

Mean maternal age did not differ between mothers of children with (29.7 years; SD 5.1) and without GBS infection (29.9 years; SD 5.1), but a higher proportion of mothers of infants with GBS disease was primiparous and had pre-eclampsia, compared to mothers of infants without infection (Table 1).

**Table 1:** Maternal and infant characteristics of 625 liveborn infants with and 1,006,621 without invasive Group B Streptococcal infection born in Norway during 1996–2012.

	GBS infection	No GBS infection	*P* value
	*N* (%)	*N* (%)	
Maternal characteristics			
Primigravida	345 (55)	415‚767 (41)	<0.001*
Chronic hypertension prior to pregnancy	5 (0.8)	5242 (0.5)	0.33*
Diabetes prior to pregnancy	10 (1.6)	6484 (0.6)	0.003*
Gestational diabetes	8 (1.3)	11,476 (1.1)	0.74*
Pre-eclampsia	47 (7.5)	37,440 (3.7)	<0.001*
Pregnancy-induced hypertension	14 (2.2)	16,341 (1.6)	0.22*
PROM^a^	168 (26.9)	112,319 (11.2)	<0.001*
PROM > 24 h^b^	87 (14.2)	52,836 (5.3)	<0.001*
Discolored/malodourous AF	124 (19.8)	157,729 (15.7)	0.004*
Mode of delivery^c^			
Elective cesarean	21 (3.4)	59,892 (5.9)	
Emergency cesarean	151 (24.2)	96,113 (9.5)	
Vaginal delivery	453 (72.5)	849,932 (84.4)	<0.001*
Induction of labor	121 (19.4)	148,745 (14.8)	
Fetal characteristics			
Prematurity^d^	194 (31.8)	65,963 (6.7)	<0.001*
Male	326 (52.2)	516,872 (51.3)	0.68*
Multiple gestation	58 (9.3)	34,721 (3.4)	<0.001*
SGA	23 (3.8)	19,588 (2.0)	0.002*
VLBW (<1500 g)	95 (15.2)	8680 (0.9)	<0.001*
Apgar 5 min			
0–3	18 (2.9)	2793 (0.3)	
4–7	49 (7.9)	9848 (1.0)	
8–10	552 (89.2)	991,387(98.7)	<0.001**
Admission to NICU	459 (73.4)	94,031 (9.3)	<0.001*
Respiratory support	105 (16.8)	5785 (0.6)	<0.001*

*GBS* Group B Streptococcus, *PROM* prelabor rupture of membranes, *AF* amniotic fluid, *SGA* small for gestational age, *VLBW* very low birth weight, *NICU* neonatal intensive care unit.

*Pearson chi square.

**Linear-by-linear association.

^a^PROM, i.e. prelabor rupture of membranes; that is, rupture of membranes before the onset of contractions.

^b^PROM > 24 h, that is, time from prelabor rupture of membranes to delivery exceeds 24 h.

^c^Six hundred and eighty-four cases of cesarean section were unspecified.

^d^Prematurity; GA < 37 weeks, information on GA missing in 14 (2.2%) GBS cases and 22 239 (2.2%) non-GBS cases.

Infants with GBS infection were more often born by an emergency cesarean section, and/or they were born 24 h or more after PROM. Although the majority of infants with GBS infection were born at term, infants with GBS infection were more likely to be born preterm (Table 1), with a mean gestational age of 36.8 weeks (SD 4.8), compared to infants without such infection (mean gestational age: 39.3 weeks; SD 2.1). In line with this difference in gestational age, the mean birth weight was lower in infants with (3016 g, SD 1107 g) than without GBS infection (3522 g, SD 607 g). Moreover, children with GBS infection were more likely to be part of multiple gestation pregnancies, to have very low birth weight, and to have lower 5-min Apgar scores, than children without GBS infection (Table 1).

Among infected children, 411 (65.8%) had EOD, 202 (32.2%) had LOD, and only 12 children (1.9%) had VLOD. EOD was particularly common among infants born at term, while among those born before 28 weeks of gestation, LOD was most common (Table 2). Sepsis was the most common diagnosis in all gestational age groups and was diagnosed in 352 (85.6%) infants with EOD, in 140 (69.3%) with LOD, and in 6 (50%) infants with VLOD. Meningitis was diagnosed in 38 (8.6%) of the cases with EOD, in 54 (26.7%) of those with LOD, and in 6 (50%) of the 12 cases with VLOD.

**Table 2:** Clinical diagnosis and age at onset of invasive Group B Streptococcal infection, stratified by gestational age (GA).

	GA ≤ 27	GA 28–36	GA ≥ 37	All GA^a^
	*N* (%)	*N* (%)	*N* (%)	*N* (%)
All GBS cases	55 (100)	139 (100)	417 (100)	625 (100)
Diagnosis				
Sepsis	47 (85.5)	121 (87.1)	316(75.8)	498 (79.7)
Meningitis	8 (14.5)	15 (10.8)	75 (18.0)	98 (15.7)
Pneumonia	0 (0)	1 (0.07)	14 (3.4)	15 (2.4)
Other^b^	0 (0)	2 (0.14)	12 (2.9)	14 (2.2)
Age at disease onset				
EOD^c^	11 (20.0)	81 (58.3)	310 (74.3)	411 (65.8)
LOD^d^	43 (78.2)	54 (38.8)	100(24.0)	202 (32.3)
VLOD^e^	1 (1.8)	4 (2.9)	7 (1.7)	12 (1.9)

^a^Information on gestational age (GA) was missing in 14 GBS cases.

^b^Other invasive infections include urinary tract infection, arthritis, and clinical diagnosis not further specified in MSIS.

^c^EOD: 0–6 days of age.

^d^LOD: 7–89 days of age.

^e^VLOD: 90–365 days.

**Fig. 3:**
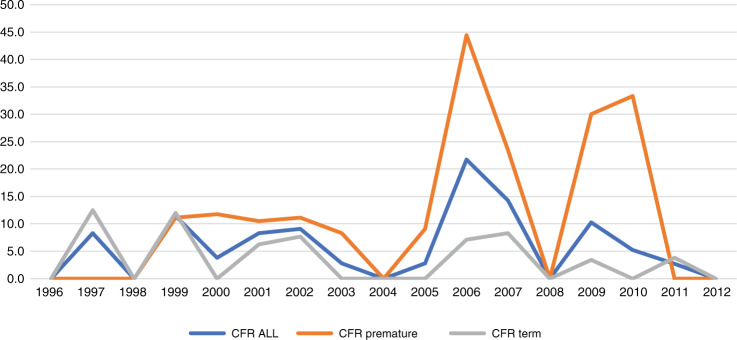
Case fatality rate (CFR). The figure shows the the overall (blue line) case fatality rate in percentage (Y-axis) of invasive Group B Streptococcal (GBS) infection during the first year of in liveborn children in Norway during 1996–2012 (X-axis) as well as the trends for premature born children (orange line), and term born children (grey line). For calculations, see Supplementary Table S2.

Of the children with invasive GBS infection, 44 (7%) died during infancy and 39 died of the disease according to the MSIS (Table 3); thus, the case fatality rate was 6.2%. The highest CFR was reported in 2006, when 10 out of 46 cases died (21.7%) (Fig. 3).

**Table 3:** One-year mortality among infants with and without invasive Group B Streptococcal infection, in the total population and among infants born preterm (i.e., before gestational week 37) or at term (gestational week 37 or later)^a^.

	One-year mortality		OR (95% CI)
	Died	Survived	
	*N* (%)^b^	*N* (%)^b^	
All children			
GBS infection	44 (7.0)	581 (93.0)	24.5 (18.0–33.3)
No GBS infection	3103 (0.3)	1,003,518 (99.7)	1.0 (ref.)
Premature			
GBS infection	27 (13.9)	167 (86.1)	6.5 (4.3–9.8)
No GBS infection	1597 (2.4)	64,366 (97.6)	1.0 (ref.)
Term			
GBS infection	17 (4.1)	400 (95.9)	28.6 (17.6–46.6)
No GBS infection	1361 (0.1)	917,058 (99.9)	1.0 (ref.)

*GBS* Group B Streptococcus.

^a^Information on gestational age was missing in 14 (2.2%) cases with and 22,239 (2.2%) cases without GBS infection.

^b^Percentages are shown in rows.

Compared with children without GBS infection, the OR for death in infancy for children with GBS infection was 24.5 (95% CI: 18.0–33.3). Although the estimated relative risk for death was highest among children born at term, the absolute risk was highest in children born preterm (Table 3).

CP was diagnosed in 24 (4.1%) of the 581 children who survived a GBS infection (Table 4), compared with 1887 (0.19%) of the children without infection (OR: 22.9; CI: 15.1–34.5). Although the estimated relative risk of CP was highest among children born at term, the absolute risk was highest in prematurely born children (Table 4). The mean gestational age among children who survived GBS in infancy and were later diagnosed with CP was 34.1 weeks (SD 6.3), compared with 37.1 weeks (SD 4.6) among survivors without CP.

**Table 4:** Risk of cerebral palsy following invasive GBS infection, in the total population and among infants born preterm (i.e., before gestational week 37) or at term (gestational week 37 or later)^a^.

	Cerebral palsy		OR (95% CI)
	Yes	No	
	*N* (%)^b^	*N* (%)^b^	
All children			
GBS infection	24 (4.1)	557 (95.9)	22.9 (15.1–34.5)
No GBS infection	1887 (0.19)	1,001,631 (99.8)	1.0 (ref.)
Premature			
GBS infection	11 (6.6)	156 (93.4)	6.1 (2.9–11.3)
No GBS infection	735 (1.1)	63,631 (98.9)	1.0 (ref.)
Term			
GBS infection	12 (3.0)	388 (97.0)	25.7 (14.4–45.7)
No GBS infection	1104 (0.12)	915,954 (99.9)	1.0 (ref.)

*GBS* Group B Streptococcus.

^a^Information on gestational age was missing in 14 (2.2%) GBS cases and 22,239 (2.2%) cases without GBS.

^b^Percentages are shown in rows.

Among 91 children surviving meningitis, CP was diagnosed in 8 (8.8%), compared with 16 of the 464 (3.4%) survivors of sepsis (*p* = 0.036). None of the children with pneumonia or other invasive diagnosis were diagnosed with CP (data not shown). Among premature born children with CP, 1.5% were diagnosed with invasive GBS infection during infancy, while among term born children with CP, 1.1% had a history of GBS infection in infancy. There were no clear differences in CP subtypes between those who had been diagnosed with invasive GBS infection, compared with children without a history of such infection (Table 5). The proportions of children with CP who had the most severe impairments was lower in the group with a history of GBS infection compared with children with CP, but without GBS infection, although none of these differences reached statistical significance (Table 5). The proportion of children with epilepsy (4.2%) was identical in children with CP associated with GBS infection and in those without a history of such infection (Table 5).

**Table 5:** CP subtype, gross and fine motor function, and associated problems in 24 children with and in 1887 without invasive GBS infection during infancy.

	GBS infection *N* (%)	No GBS infection *N* (%)	*P* value
CP subtype			
Spastic unilateral	11 (45.8)	783 (41.5)	
Spastic Bilateral	9 (37.5)	856 (45.4)	
Dyskinetic	2 (8.3)	122 (6.5)	
Ataxic	2 (8.3)	81 (4.3)	
Nonclassified CP	0 (0)	43 (2.3)	0.74*
GMFCS			
I and II	17 (81.0)	1077 (68.3)	
III	2 (9.5)	115 (7.3)	
IV and V	2 (9.5)	384 (24.4)	0.14**
MACS			
I and II	16 (84.2)	973 (68.1)	
III	2 (10.5)	170 (11.9)	
IV and V	1 (5.3)	285 (20.0)	0.097**
VIKING^a^			
I and II	14 (70.6)	1086 (66.8)	
III	2 (11.8)	136 (8.4)	
IV	3 (17.6)	400 (24.8)	0.43**
Epilepsy	1 (4.2)	82 (4.3)	1.00***
Gastrostomy	1 (4.2)	221 (11.7)	0.30***

*GBS* Group B Streptococcus, *GMFCS* gross motor function classification system, *MACS* Manual Ability Classification System.

*Pearson chi square.

**Linear-by-linear association.

***The unconditional z-pooled test.

^a^VIKING Speech scale.

In the sensitivity analyses, children who had positive cultures and a specified clinical diagnosis were included in the case group (*N* = 591), while children whose diagnoses were based on a positive GBS urine antigen test (*N* = 21), or GBS isolated from lung aspirate (*N* = 1), unknown (*N* = 3), or other material (*N* = 9) were added to the control group. The results were essentially unchanged (data not shown).

## Discussion

In this population-based study, we found that the incidence of invasive GBS infection was 0.62 per live births in Norway. While the incidence of EOD and VLOD remained stable during the study period, the incidence of LOD increased. In line with our hypothesis, GBS infections were associated with a considerably increased risk of death and CP compared with children without such infection. The 1-year mortality was 7%, while the case fatality rate was 6.2%. In contrast to our hypothesis, there was a tendency towards milder motor impairments in children who had been diagnosed with a GBS infection, compared with children without such infection. Moreover, we could not confirm the hypothesis that bilateral CP was more common.

Strengths of the present study are the large number of births and the prospective recording of data in the MBRN, the CPRN, and the MSIS. The main findings are unlikely to be caused by chance as indicated by the 95% confidence intervalls. We report both 1-year mortality and case fatality rates, since there may be underreporting of case fatality rates to the MSIS. It is therefore reassuring regarding the validity of our results that the 1-year mortality and the case fatality rates were similar.

The correctness of the CP diagnosis in the CPRN at 5 years of age, and the unbiased selection of cases as documented in a validation study are further strengths.^13^ A potential misclassification of a few children with CP as controls is negligible compared to the 1,007,246 children comprising the latter group.

A limitation of the present study is the lack of information on maternal antibiotic treatment and suspected maternal infection (i.e., fever, or clinical chorioamnionitis). Moreover, restricting the diagnosis to infants with laboratory documented GBS infection is likely to underestimate the number of infected infants, and thus the true incidence of the disease. Whereas prophylactic treatment is likely to prevent invasive infection, it is unclear if the prognosis of children with invasive GBS infection, but without a positive culture, differs considerably from those with positive cultures.^22^

Since both CP and GBS infections are rare disorders, the number of children included in the subgroup analyses for CP subtypes and associated problems are relatively low, resulting in limited statistical power. Thus, some of the subgroup analyses must be interpreted with caution, in particular the findings regarding CP subtypes and motor impairments.

The incidence of invasive GBS infection during the first year of life was 0.62 per 1000 live births in this study. The incidences of EOD (0.41 per 1000 live births) and of LOD (0.20 per 1000 live births) in our study are in keeping with the results of a recent meta-analysis reporting an incidence of 0.37 (95% CI: 0.30–0.44) for EOD and of 0.21 (95% CI: 0.16–0.26) for LOD in high-income countries.^1^ Increasing incidences of LOD have also been reported in recent studies from Spain, France, Greece, and the UK.^8–11^ While one study suggested that the increased incidence of LOD was associated with an increasing proportion of infants being born premature,^9^ this is unlikely to explain the observed increase in our study, since the prevalence of prematurely born children in Norway was stable during the study period.^14^

The case fatality rate in our study (6.2%) was slightly higher than the average rate of 4.7% reported in high-income countries according to a recent meta-analysis.^1^ However, the CFR in our study was 5.0% when cases diagnosed during 2006 were excluded. A survey following the peak in CFR in Norway during 2006 did not reveal a single explanation, but suggested that a more virulent serotype V could explain some of the fatal cases.^23^ Thus, the increasing LOD incidence in Norway might be explained by a shift in serotype and virulence profile of the strains, but further studies are warranted to clarify this.

Among survivors of invasive GBS infection, 4.1% were later diagnosed with CP. The highest proportion (8.8%) was observed among children who had meningitis. In the above-mentioned meta-analysis, 18 studies assessed long-term neurodevelopmental impairments (NDI).^1^ They found that among survivors of meningitis, 18% (95% CI: 13–22%) had moderate to severe NDIs at 18 months follow-up. However, except for GBS meningitis, the authors of the meta-analysis were unable to calculate OR for NDI following other invasive GBS infections, given the available data. CP as a specific outcome of invasive GBS infection was not evaluated in the meta-analysis.^1^ A recent study from Australia reported that only 1.2% of survivors of GBS infection were diagnosed with CP.^24^ In that study, the mortality within 11 years of age was only 3.0%, compared with a 7.0% 1-year mortality in our study. The numbers of included infants in the two studies were comparable; however, it may be noteworthy that the incidence of GBS infection in the Australian study (1.0 per 1000) was considerably higher than in our study (0.62 per 1000), and more than twice the incidence reported in the recent meta-analysis (based on facility studies only) of 0.46 per 1000 live births.^1,24^ Thus, the different results may be explained by different diagnostic criteria for GBS infection, different serotype and virulence profile of the GBS strains, differences in the application of intrapartum antibiotic prophylaxis (IAP) and demographic differences between the populations studied.^24^

The mortality of invasive GBS infection in infants, in particular in preterm born infants, and the high proportion of survivors later diagnosed with CP is likely to be explained by the functional immature immune system of the infant.^25^ Several factors involved in invasive infection may lead to death or to NDI in survivors. These factors include inflammatory cytokines (inflammation), disseminated intravascular coagulation and hypotension, and a sensitizing effect due to bloodstream infection, leading to development of hypoxic–ischemic encephalopathy.^26–28^ When these mechanisms lead to injuries or damages to the motor cortex or basal ganglia of the immature brain, they may cause CP.^29,30^ Other potential complications of GBS meningitis that may lead to CP are arterial stroke, sinus venous thrombosis,^31,32^ and hydrocephalus.^33^ It is therefore also reasonable that CP was most commonly a complication of meningitis. As opposed to our hypothesis, children with CP who were survivors of invasive GBS infection did not have more severe motor impairments than children with CP of other causes. Thus, we speculate that CP associated with invasive GBS infection is not caused by a specific pathophysiological process, but similar to the general processes thought to cause CP, a GBS infection may initiate a continuum of processes from inflammation, via circulatory collapse to severe hypoxia-ischemia.^34^

Although the incidence of EOD in our study was within the range reported in the recent meta-analysis, incidences below 0.25 have been reported in both countries practicing screening- and risk-based strategies.^6,35,36^ Thus, it may be discussed if the current risk-based guidelines could be further refined, or if screening-based IAP should be considered in Norway.

While there is no international consensus regarding prevention strategy of EOD GBS disease, most countries recommend IAP during preterm labor (i.e., GA < 37), regardless of colonization status.^37^ In contrast, in Norway IAP is only administered in preterm births (<37 weeks) if maternal colonization of GBS has been confirmed, and not if GBS status is unknown. While a swab is taken in case of preterm labor, the result is often unknown at the time of delivery. In Denmark, IAP during labor is recommended if gestational age is <35 weeks, regardless of maternal GBS status. Whether the differences in incidence between the present study population and the Danish population are due to differences in prevention guidelines cannot be answered by this study.

Moreover, many countries treat women with term PROM (>18 h) with antibiotics if proven GBS colonization or unknown GBS status.^5,37^ In Norway, IAP is only administered if there are signs of infection, or if colonization is detected by chance in deliveries where PROM exceeds 18 h, while induction of labor is indicated 24 h after PROM.^38^ Because extensive use of antibiotics might have serious short- and long-term consequences, it can be debated if applying IAP in all cases of PROM to prevent some GBS cases is appropriate. If IAP is applied in all cases of PROM >18 h, 8% of women giving birth at term will receive IAP; moreover, the RCOG guidelines suggested that in this case the number needed to treat to prevent one death caused by invasive GBS infection in term born children would be >10,000.^38,39^

Another important issue in the prevention of GBS infections is whether/how/if clinicians adhere to the different guidelines. In a Swedish national GBS prevalence study, only 14% of cases with risk factors received IAP in line with the guidelines.^40^ In that study, 40% of pregnant women with GBS-infected children had no risk factors for neonatal GBS disease. However, in cases where the infant died of a GBS infection, at least one risk factor was identified, and IAP had not been administered during delivery in any of those cases.^40^ Also, two small studies from Norway suggested lack of compliance to guidelines.^41,42^ Thus, there is probably a potential for improving the outcome of invasive GBS infection by stricter adherence to national guidelines. Although we were not able to address this issue in our study, better implementation of existing guidelines for IAP, and more precise guidelines, could possibly reduce the incidence of EOD further. Due to concerns regarding antibiotic resistance and disruption of the establishment of the newborn microbiota,^7^ it seems reasonable to first ensure that targeted treatment is optimized before opting for a screening-based strategy.

Another option is to improve targeted prophylaxis by identifying particularly virulent strains, screening all women and treating only those colonized with these particular strains. While colonizing GBS strains are considered less virulent than invasive strains,^43^ further in-depth studies are needed to identify virulent strains with increased ability to cause invasive infection and adverse outcomes.

In contrast to the incidence of EOD, the incidence of LOD is unlikely to be affected by IAP.^44^ LOD may develop through nosocomial transmission in hospitalized infants, by horizontal transmission, or through vertical transmission from mother to infant via skin or breastmilk. However, in most cases, the source of transmission remains unknown, which makes it difficult to implement preventive measures. The frequency of nosocomial outbreaks is presumably low.^11^ Nonetheless, such outbreaks do occur, and adherence to infection control guidelines is required to prevent this.

One potential strategy to reduce both early- and late-onset GBS disease is maternal vaccination. Vaccination is expected to induce high levels of GBS-specific immunoglobulin G (IgG) in the mother. While IgG is able to cross the placenta, the majority of fetal IgG is acquired during the last 4 weeks of pregnancy.^45^ Efficacy of maternal vaccination might therefore be less effective in preventing GBS disease in infants born preterm, as well as in preventing stillbirths at low gestational ages. However, it has been proposed that an effective vaccine could reduce mucosal colonization, produce both humoral and mucosal immunity, and thereby prevent both LOD and EOD.^46^ Several vaccines are under development, and trials have shown them to be safe and immunogenic. Phase 3 trials have not been completed, and it is not known when a safe and immunogenic vaccine will be licensed.^47^

## Conclusion

Despite a relatively low incidence of invasive GBS infection in Norwegian infants, the burden of such infections remains high. Preterm infants are at high risk of both death and CP. While the burden of EOD probably can be further reduced by better targeted guidelines and better adherence to these guidelines, the burden of disease among premature children might indicate the need for updating guidelines to include premature birth as a risk factor, in line with guidelines from The Royal College of Obstetricians and Gynecologists. A discussion of existing prevention guidelines and how to ensure better adherence is needed. The burden of LOD and VLOD is likely not affected by updating these guidelines, and will most likely depend on an effective vaccine. Further studies of the increasing LOD incidence is needed, as well as studies on the role of virulence of GBS in relation to mortality and long-term neurological impairments.

## Supplementary information

Supplementary table S1, table S2
