# Correction: Incidence of invasive Group B Streptococcal infection and the risk of infant death and cerebral palsy: a Norwegian Cohort Study

**DOI:** 10.1038/s41390-021-01887-8

**Published:** 2021-12-09

**Authors:** Maren Mynarek, Solveig Bjellmo, Stian Lydersen, Jan E. Afset, Guro L. Andersen, Torstein Vik

**Affiliations:** 1 Department of Clinical and Molecular Medicine, Norwegian University of Science and Technology, 7491 Trondheim, Norway; 2 Department of Obstetrics and Gynecology, Helse More og Romsdal HF, Aalesund, Norway; 3 Regional Centre for Child and Youth Health and Child Welfare, Department of Mental Health, PB 8905, MTFS, 7491 Trondheim, Norway; 4 Department of Medical Microbiology, St. Olavs Hospital, Trondheim University Hospital, Trondheim, Norway; 5 Vestfold Hospital Trust, The Cerebral Palsy Registry of Norway, PB 2168, 3103 Tønsberg, Norway

Correction to: *Pediatric Research* 10.1038/s41390-020-1092-2, published online 29 July 2020

The article “Incidence of invasive Group B Streptococcal infection and the risk of infant death and cerebral palsy: a Norwegian Cohort Study,” written by Maren Mynarek, Solveig Bjellmo, Stian Lydersen, Jan E. Afset, Guro L. Andersen, and Torstein Vik was originally published Online First without Open Access. After publication in volume 89, issue 6, page 1541–1548, the author decided to opt for Open Choice and to make the article an Open Access publication. Therefore, the copyright of the article has been changed to © The Author(s) 2020 and the article is forthwith distributed under the terms of the Creative Commons Attribution 4.0 International License, which permits use, sharing, adaptation, distribution, and reproduction in any medium or format, as long as you give appropriate credit to the original author(s) and the source, provide a link to the Creative Commons licence, and indicate if changes were made.

The images or other third-party material in this article are included in the article’s Creative Commons licence, unless indicated otherwise in a credit line to the material. If material is not included in the article’s Creative Commons licence and your intended use is not permitted by statutory regulation or exceeds the permitted use, you will need to obtain permission directly from the copyright holder.

