# Supplementary material for: Incidence of invasive Group B Streptococcal infection and the risk of infant death and cerebral palsy: a Norwegian Cohort Study
Source: Pediatr Res. 2020 Jul 29;89(6):1541–8. doi: 10.1038/s41390-020-1092-2 (PMC8660635; doi:10.1038/s41390-020-1092-2)
Supplement: Supplementary file 1 — Supplementary table S1, table S2 [file 41390_2020_1092_MOESM1_ESM.docx]

| **Supplementary table S1: Incidence of all invasive Group B streptococcal infections (GBS), early onset disease (EOD), late onset disease (LOD) and very late onset disease (LOD) among live born children in Norway the respective year.** | | | | | |
| --- | --- | --- | --- | --- | --- |
| **Birth year** | **Invasive GBS infection** | **Live births** | **Incidence per 1000** | **95 % Confidence interval** | |
| **Incidence all** | |  |  |  |  |
| 1996 | 18 | 60588 | 0.30 | (0.19 to 0.47) | |
| 1997 | 36 | 59430 | 0.61 | (0.44 to 0.84) | |
| 1998 | 32 | 57967 | 0.55 | (0.39 to 0.78) | |
| 1999 | 35 | 59330 | 0.59 | (0.42 to 0.82) | |
| 2000 | 52 | 59294 | 0.88 | (0.67 to 1.15) | |
| 2001 | 36 | 56818 | 0.63 | (0.46 to 0.88) | |
| 2002 | 22 | 55884 | 0.39 | (0.26 to 0.60) | |
| 2003 | 36 | 56704 | 0.63 | (0.46 to 0.88) | |
| 2004 | 30 | 57456 | 0.52 | (0.37 to 0.75) | |
| 2005 | 36 | 57198 | 0.63 | (0.45 to 0.87) | |
| 2006 | 46 | 58940 | 0.78 | (0.59 to 1.04) | |
| 2007 | 42 | 58898 | 0.71 | (0.53 to 0.96) | |
| 2008 | 43 | 61122 | 0.70 | (0.52 to 0.95) | |
| 2009 | 39 | 62693 | 0.62 | (0.46 to 0.85) | |
| 2010 | 38 | 62312 | 0.61 | (0.44 to 0.84) | |
| 2011 | 37 | 61071 | 0.61 | (0.44 to 0.83) | |
| 2012 | 47 | 61114 | 0.77 | (0.58 to 1.02) | |
|  |  |  |  |  |  |
| **Incidence EOD** | | | |  |  |
| 1996 | 14 | 60588 | 0.23 | (0.14 to 0.39) | |
| 1997 | 26 | 59430 | 0.44 | (0.30 to 0.64) | |
| 1998 | 21 | 57967 | 0.36 | (0.24 to 0.55) | |
| 1999 | 24 | 59330 | 0.40 | (0.27 to 0.60) | |
| 2000 | 44 | 59294 | 0.74 | (0.55 to 1.00) | |
| 2001 | 23 | 56818 | 0.40 | (0.27 to 0.61) | |
| 2002 | 15 | 55884 | 0.27 | (0.16 to 0.44) | |
| 2003 | 28 | 56704 | 0.49 | (0.34 to 0.71) | |
| 2004 | 23 | 57456 | 0.40 | (0.27 to 0.60) | |
| 2005 | 19 | 57198 | 0.33 | (0.21 to 0.52) | |
| 2006 | 26 | 58940 | 0.44 | (0.30 to 0.65) | |
| 2007 | 26 | 58898 | 0.44 | (0.30 to 0.65) | |
| 2008 | 24 | 61122 | 0.39 | (0.26 to 0.58) | |
| 2009 | 25 | 62693 | 0.40 | (0.27 to 0.59) | |
| 2010 | 29 | 62312 | 0.47 | (0.32 to 0.67) | |
| 2011 | 20 | 61071 | 0.33 | (0.21 to 0.51) | |
| 2012 | 24 | 61114 | 0.39 | (0.26 to 0.58) | |
| **Incidence LOD** | | |  |  |  |
| 1996 | 4 | 60588 | 0.07 | (0.03 to 0.17) | |
| 1997 | 9 | 59430 | 0.15 | (0.08 to 0.29) | |
| 1998 | 7 | 57967 | 0.12 | (0.06 to 0.25) | |
| 1999 | 10 | 59330 | 0.17 | (0.09 to 0.31) | |
| 2000 | 8 | 59294 | 0.13 | (0.07 to 0.27) | |
| 2001 | 13 | 56818 | 0.23 | (0.13 to 0.39) | |
| 2002 | 7 | 55884 | 0.13 | (0.06 to 0.26) | |
| 2003 | 8 | 56704 | 0.14 | (0.07 to 0.28) | |
| 2004 | 6 | 57456 | 0.10 | (0.05 to 0.23) | |
| 2005 | 15 | 57198 | 0.26 | (0.16 to 0.43) | |
| 2006 | 19 | 58940 | 0.32 | (0.21 to 0.50) | |
| 2007 | 15 | 58898 | 0.25 | (0.15 to 0.42) | |
| 2008 | 19 | 61122 | 0.31 | (0.20 to 0.49) | |
| 2009 | 14 | 62693 | 0.22 | (0.13 to 0.37) | |
| 2010 | 9 | 62312 | 0.14 | (0.08 to 0.27) | |
| 2011 | 16 | 61071 | 0.26 | (0.16 to 0.43) | |
| 2012 | 23 | 61114 | 0.38 | (0.25 to 0.56) | |
| **Incidence VLOD** | | |  |  |  |
| 1996 | 0 | 60588 | 0.00 | (0.00 to 0.06) | |
| 1997 | 1 | 59430 | 0.02 | (0.00 to 0.10) | |
| 1998 | 4 | 57967 | 0.07 | (0.03 to 0.18) | |
| 1999 | 1 | 59330 | 0.02 | (0.00 to 0.10) | |
| 2000 | 0 | 59294 | 0.00 | (0.00 to 0.06) | |
| 2001 | 0 | 56818 | 0.00 | (0.00 to 0.07) | |
| 2002 | 0 | 55884 | 0.00 | (0.00 to 0.07) | |
| 2003 | 0 | 56704 | 0.00 | (0.00 to 0.07) | |
| 2004 | 1 | 57456 | 0.02 | (0.00 to 0.10) | |
| 2005 | 2 | 57198 | 0.03 | (0.01 to 0.13) | |
| 2006 | 1 | 58940 | 0.02 | (0.00 to 0.10) | |
| 2007 | 1 | 58898 | 0.02 | (0.00 to 0.10) | |
| 2008 | 0 | 61122 | 0.00 | (0.00 to 0.06) | |
| 2009 | 0 | 62693 | 0.00 | (0.00 to 0.06) | |
| 2010 | 0 | 62312 | 0.00 | (0.00 to 0.06) | |
| 2011 | 1 | 61071 | 0.02 | (0.00 to 0.09) | |
| 2012 | 0 | 61114 | 0.00 | (0.00 to 0.06) | |

| **Supplementary table S2: Case fatality rate (CFR) among children with invasive Group B Streptococcal infection in infancy, calculated as number of children that died of the disease, divided by the number of infants who were diagnosed with the disease the respective year**. | | | |
| --- | --- | --- | --- |
| **Birth year** | **CFR ALL^a^** | **CFR Premature^b^** | **CFR Term^c^** |
| 1996 | 0 % (0/18) | 0 % (0/7) | 0 % (0/10) |
| 1997 | 8.3 % (3/36) | 0 % (0/8) | 12.5 % (3/24) |
| 1998 | 0% (0/32) | 0 %(0/8) | 0 % (0/21) |
| 1999 | 11.4 % (4/35) | 11.1 % (1/9) | 12.0 % (3/25) |
| 2000 | 3.8 % (2/52) | 11.8 % (2/17) | 0 % (0/35) |
| 2001 | 8.3 % (3/36) | 10.5 % (2/19) | 6.3 % (1/16) |
| 2002 | 9.1 % (2/22) | 11.1 % (1/9) | 7.7 %(1/13) |
| 2003 | 2.8 % (1/36) | 8.3 % (1/12) | 0 % (0/24) |
| 2004 | 0 % (0/30) | 0 % (0/7) | 0 % (0/21) |
| 2005 | 2.8 %(1/36) | 9.1% (1/11) | 0 % (0/25) |
| 2006 | 21.7 % (10/46) | 44.4% (8/18) | 7.1 % (2/28) |
| 2007 | 14.3 % (6/42) | 23.5 % (4/17) | 8.3 % (2/24) |
| 2008 | 0 % (0/43) | 0 % (0/13) | 0 % (0/29) |
| 2009 | 10.3 % (4/39) | 30 % (3/10) | 3.4 % (1/29) |
| 2010 | 5.3 % (2/38) | 33.3 % (2/6) | 0 % (0/32) |
| 2011 | 2.7 % (1/37) | 0 % (0/11) | 3.8 % (1/26) |
| 2012 | 0 % (0/47) | 0 % (0/12) | 0 % (0/35) |
| ^a^ Information on gestational age missing among 1 child diagnosed in 1996, 4 in 1997, 3 in 1998, 1 in 1999, 1 in 2001, 2 in 2004, 1 in 2007 and 1 in 2008.  ^b^ Premature; GA <37 weeks of gestation,  ^c^ Term; GA ≥37 weeks of gestation | | | |
